# Choroidal metastasis from carcinoma of breast detected on F18-FDG PET CT scan: A case report and review of literature

**DOI:** 10.4103/0972-3919.78252

**Published:** 2010

**Authors:** Shrikant Solav, Ritu Bhandari, Anuradha Sowani, Sameer Saxena

**Affiliations:** Spect Lab Nuclear Medicine Services, Kothrud, Pune, India

**Keywords:** Distant metastasis, paranasal sinuses, thyroid neoplasms

## Abstract

Intraocular choroidal metastasis is a very rare cause of blindness. Choroidal hemangioma and melanoma are other causes that may mimic the condition. Carcinoma of breast is the most common primary malignancy that accounts for choroidal metastasis in females and carcinoma of lung is the most common cause in males. Other primary neoplasms which can uncommonly metastasize to the choroid are testis, gastrointestinal tract, kidney, thyroid, pancreas, and prostate. Metastatic neoplasm to the eye outnumbers the primary tumors such as retinoblastomas and malignant melanoma. Sonography is usually the initial investigation after fundus examination to look for the architecture of the lesion. However, it lacks in specificity. We present a case of carcinoma of breast that had visual disturbances and wholebody F18-fluorodeoxyglucose, positron emission tomography-computerized tomography (FDG PET CT) revealed a choroidal lesion in addition to cerebral, pulmonary, and skeletal metastases. Choroidal metastasis from carcinoma of lung has been reported previously on FDG PET. To the best of our knowledge, this is the first case report of carcinoma of breast demonstrating choroid metastasis on F18-FDG PET CT scan.

## INTRODUCTION

Carcinoma of breast is one of the most common malignancies in women, with a lifetime risk of approximately 13%. The diagnosis is done by self examination or screening mammography. Fluorine 18-Fluorodeoxyglucose–positron emission tomography-computerized tomography (F18-FDG-PET CT) can detect the primary with a sensitivity of 79 to 90%. Its sensitivity is 80 to 95% for detection of distant metastasis. The negative predictive value of FDG PET CT is 90%. Metastasis can occur at any location. Axillary lymph nodes, internal mammary lymph nodes, bone, liver, and brain are the organs involved in order of frequency.[1] Intraocular choroidal metastasis is a rare metastatic site. Although among all the primary tumors, carcinoma of breast is the most common cause for choroidal metastasis, an incidence of 9% has been reported for choroidal metastases in asymptomatic patients who had metastatic breast carcinoma.[2] The average interval between the diagnosis of the primary tumor and that of uveal metastases has been reported to be 3 to 6 years.[3] In the present case, there was an interval of 9 years between initial diagnosis and manifestation of ophthalmic involvement.

## CASE REPORT

**Figure 1:**
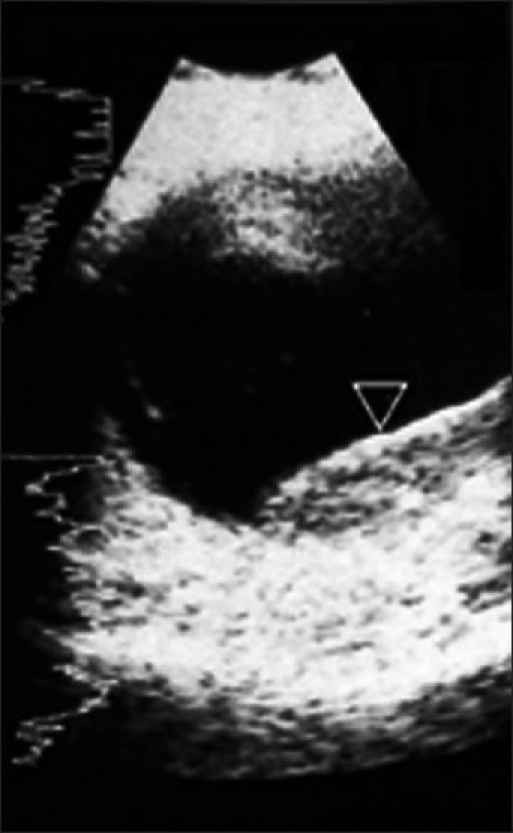
Ultrasonography of left eye shows a well-defined, echogenic lentiform-shaped lesion along the temporal aspect of the posterior coat of the globe associated with retinal detachment (►). A differential diagnosis of melanoma, metastasis was made

**Figure 2:**
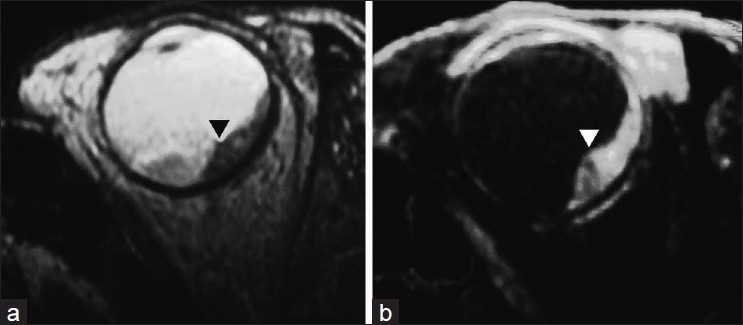
Magnetic resonance imaging shows a plaque-like lesion of uneven thickness along the posterolateral surface of the globe in close relation to the choroid (►) with associated retinal detachment. The lesion is hypointense on T2W study and hyperintense on STIR images

**Figure 3:**
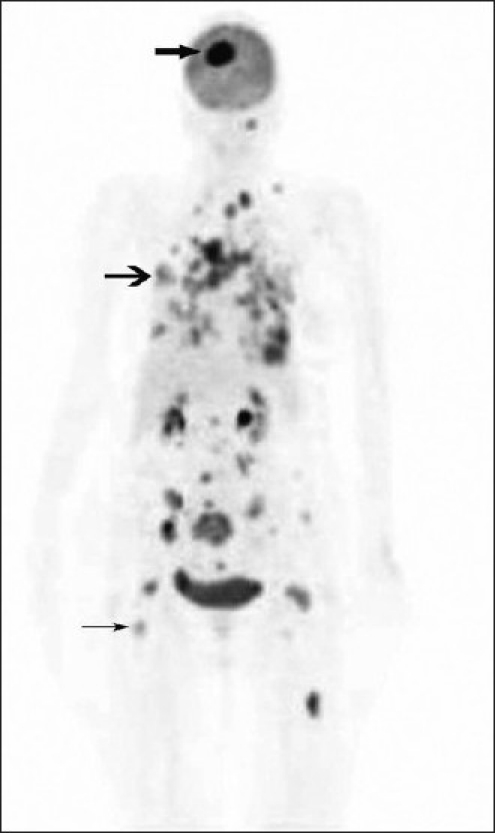
F18-FDG PET CT study: Maximum intensity projection image shows FDG-avid lesions in the brain (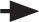), lungs (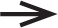), mediastinal-retroperitoneal lymph nodes, and skeleton (→)

**Figure 4:**
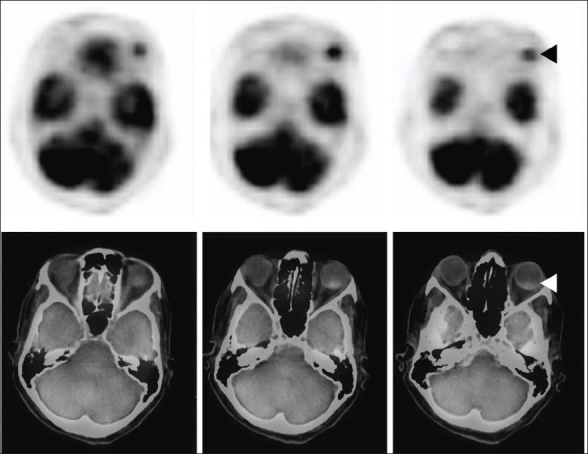
F18 FDG PET CT: Transaxial section across the orbit shows intense focus of FDG activity in the left eye ball posteriorly—fusion image shows coregistration in the posterior choroid corresponding to the radiologic lesion seen in computerized tomogram (◄)

This 70-year-old woman was treated for carcinoma of breast in 2001. She received radiation and 5 years of tamoxifen therapy. She developed hemiplegia in 2007, from which there was complete recovery. There was diminution of vision in the left eye in August 2010. Ultrasonography revealed echogenic lesion in relation to the choroid of left eye, raising a possibility of choroidal melanoma [Figure 1 open arrowhead]. An MRI (magnetic resonance imaging) of the eye ball revealed lentiform-shaped lesion along the posterolateral surface of left eye in close proximity to the choroid with associated retinal detachment [Figure 2a and b, arrowheads]. A whole-body F18-FDG PET CT scan was requested for restaging. 10 millicurie of F18-FDG was administered on 6 hours fasting state. An uptake time of 70 minutes was allowed. Whole-body PET CT images were acquired on dedicated Biograph duo system. Images were reconstructed in short axis, vertical long axis, and horizontal long axis views. Maximum intensity projection images [Figure 3: MPI] show multifocal metabolically active lesions involving the brain, mediastinal, retroperitoneal lymph nodes, lungs, and bones. Transaxial view of the eyes revealed a focus of avid FDG localization in the left choroid [Figure 4 arrowhead].

## DISCUSSION

Ocular tumors may be primary or metastatic. Metastatic ocular neoplasms outnumber the primary tumors.

Ocular metastasis usually strikes the choroid, layers of blood vessels that nourish the back of the eye. The choroid is the most vascular tissue in the eye supplied by about 20 short and long posterior ciliary arteries. Metastasis to the eye may also affect retina, iris, and optic nerve. The most common site of uveal metastasis from breast carcinoma is the choroid (85%) followed by iris (3%) and ciliary body (<1%).[4] Choroidal metastasis usually develops late in the disease. Lung and brain metastasis ordinarily precede ocular involvement. Metastatic ocular neoplasm usually arises from breast in woman and bronchus in men. Less common primary sites are the testis, gastrointestinal tract, thyroid, kidney, pancreas, carcinoid tumor, choriocarcinoma, salivary gland carcinoma, gingival carcinoma, carcinoma of cervix, ovary, endometrium, urinary bladder, and prostate. At the time of initial ocular presentation, 30% of patients may have no known primary.[5] Choroidal metastasis may be single or multiple. Sonography shows diffuse choroidal thickening with high or medium amplitude echoes and may mimic amelanotic melanoma.

Ultrasonography provides local extent of the lesion, although it cannot reliably differentiate between the primary neoplasm and metastasis. Choroidal metastasis has been reported using F18-FDG PET CT in carcinoma of lung earlier.[6] However, in the review of literature, we could not find any reference reporting choroidal metastasis from carcinoma of breast on FDG-PET CT, despite the fact that it is the most common cause of choroidal metastasis. Loss of vision does not occur unless complicated by retinal detachment. Complications of choroidal metastases include retinal detachment, which may occur in up to 90% of patients, and, rarely, massive subretinal or intravitreal hemorrhage.[7]

Primary neoplasms include malignant choroidal melanoma and retinoblastoma.

Malignant melanoma is the commonest intraocular neoplasm. 85% of ocular melanomas arise in choroid and 15% in the ciliary body.[8] These tumors are usually single or unilateral. Sonography usually shows lenticular-shaped mass in the choroid. Some melanomas may have mushroom appearance because of breach in Bruch’s membrane, leading to formation of a waist. Ciliary body melanomas are usually difficult to identify without reasonably dilated pupil. Choroidal melanomas need to be differentiated from Fuchs spots. These are post-neovascularization lesions that develop in choroidal defects as a result of traumatic or degenerative disease, leading to subretinal space bleed.[9] Choroidal melanomas may metastasize to the liver, bone, and lymph nodes. F18-FDG PET CT may be used to detect these lesions.[10] Carbon-11-Methionone has been used to evaluate the efficacy of ion beam radiotherapy in choroidal melanoma.[11] In one study, F18-FDG PET was found to be insensitive as compared with N-isopropyl-P-I123-Amphetamine in detection of uveal melanomas.[12]

Sonographic study has been done in the past to differentiate malignant melanoma from choroidal metastasis.[13] However, it is not a specific method to differentiate the two. In the present case, sonographic appearance had raised a possibility of choroidal melanoma, although the clinical profile and FDG study clearly suggested choroidal metastasis.

Melanoma may be complicated by intraocular hemorrhage, either subretinal or into the vitreous compartment. This may complicate the sonographic appearance.[14]

Retinoblastoma is the most common intraocular tumor in children. It comprises 30% of all ocular malignancies. It may be sporadic or inherited as an autosomal dominant trait in about 6% of cases. Bilateral or multicentric disease is common in the latter. Leukocoria is the usual presenting sign. Loss of normal red reflex of retina is replaced by white or grey color. Calcium deposits are commonly present and aid in diagnosis on computerized tomography scan.[15] It is important to recognize invasion of optic nerve on magnetic resonance imaging. Presently, there is no clinical role for F18-FDG PET CT in evaluation or follow-up of retinoblastoma, but a preliminary study by Moll *et al*.[16] showed that FDG PET CT allows detection of new retinoblastomas and it is feasible to use PET to evaluate recurrence in treated patients.

Enucleation is the treatment of choice followed by radiation. Optic nerve invasion is the most significant prognostic factor. Survivors are also at risk for other malignancies, in particular osteogenic sarcoma.[17,18]

The differential diagnosis includes benign lesions such as hemangiomas and inflammatory granulomas.

F18-FDG PET CT is a sensitive method to localize choroidal lesions. It may not differentiate between choroidal melanoma, retinoblastoma, and metastasis. However, in a known case of carcinoma of breast with multiorgan involvement such as here, the study virtually clinches the diagnosis of intraocular metastasis.
